# Factors associated with involuntary hospitalisation for psychiatric patients in Switzerland: a retrospective study

**DOI:** 10.1186/s12888-018-1966-6

**Published:** 2018-12-29

**Authors:** Benedetta Silva, Philippe Golay, Stéphane Morandi

**Affiliations:** Social Psychiatry Section, Community Psychiatry Service, Department of Psychiatry, Lausanne University Hospital (CHUV), Place Chauderon 18, 1003 Lausanne, Switzerland

**Keywords:** Psychiatry, Coercion, Risk factors, Inpatient

## Abstract

**Background:**

Despite the scarce evidence for patients’ benefits of coercion and its well-documented negative effects, the use of compulsion is still very common around Europe, with important variations among different countries. These variations have been partially explained by the different legal frameworks, but also by several individual-related, system-related and area-related characteristics, identified as predictors of the use of coercive measures. This study aimed to compare the socio-demographic and clinical profile as well as the referral and hospitalisation process of people voluntarily and involuntarily hospitalized in order to identify which factors could be associated with the use of coercion.

**Methods:**

All psychiatric admissions occurred between the 1st January 2015 and the 31st December 2015 were included in this retrospective study (*n* = 5027). The whole sample was split into two subgroups accordingly to the hospitalisation legal status at admission (voluntary vs involuntary) and differences between the two groups were examined. In order to identify the factors associated with coercion, all the variables reaching a *p* < .01 level of significance when comparing the two groups were included as independent variables into a multivariate logistic regression model.

**Results:**

Globally, 62% of the admissions were voluntary and 38% were involuntary. Compared to the voluntary group, involuntary patients were significantly older, more frequently widowed and living in one specific district, and had a main diagnosis of schizophrenia (F20-F29) or organic mental disorders (F00-F09). People affected by organic mental disorders (F00-F09), with higher levels of psychotic symptoms, aggression and problems with medication adherence, were more likely to be involuntarily admitted. Moreover, living in District 1, being referred by a general practitioner, a general hospital or a psychiatric hospital and being involuntarily admitted during the previous 12 months, was associated with a higher risk of coercion.

**Conclusions:**

This study identified several individual-related, as well as system-related factors associated with the use of coercion. These results allowed us to trace a clearer profile of high-risk patients and to provide several inputs that could help local authorities, professionals and researchers to develop better-targeted alternative interventions reducing the use of coercion.

**Electronic supplementary material:**

The online version of this article (10.1186/s12888-018-1966-6) contains supplementary material, which is available to authorized users.

## Background

Despite the scarce evidence for patients’ benefits of coercion [1] and its well-documented negative effects [2–5], the use of coercion is still very common and even rising all around Europe [6, 7], with important variations among countries and also within the same country. The rates of involuntary hospitalisations in Europe may range from 3 to 30% of total psychiatric admissions [8]. These variations have been partially explained by the different legal frameworks in place within each country [9], but also by several individual-related, system-related and area-related characteristics, identified as predictors of the use of coercive measures.

At the individual level, being a young man, homeless, unemployed and affected by schizophrenia or by an organic mental disorder seems to increase the risk of being involuntarily treated [1, 10–17].

At the system level, referral procedures such as having a contact with the police or being referred by someone who does not know the patient increase the probability of being hospitalised under compulsion [18]. In Netherlands, a significant reduction of involuntary hospitalisation rate was registered following the development of a partnership between local police, social and health services [19]. In 2006, in the Canton of Geneva, Switzerland, the proportion of involuntary hospitalisations also decreased significantly after restricting the authorization to require compulsory admission to certified psychiatrists [11]. On the contrary, the link between the evolution of involuntary admission rates and the variations in the number of psychiatric beds is still unclear [20]. While some studies found that the annual reduction in provision of psychiatric beds was significantly associated with increased rates of involuntary admission [21], others highlighted that this association was rather weak and that future research should also take the influence of other service system characteristics into account [22].

Finally, at the area level, several studies have shown that living in an urban area, socio-economically deprived and with a high proportion of young adults (20–39 years) and ethnic minorities increases the risk of involuntary hospitalisation [14, 21, 23].

Switzerland has one of the highest involuntary admission rates in Europe (1.7 per 1′000 inhabitants in 2016), with significant intercantonal variations ranging from 3.3 for the Canton of Vaud to 0.4 for the Canton of Valais [24]. Over the last few years, the local authorities have set an explicit goal of limiting as much as possible the use of compulsory admission, especially within the cantons where the rates of involuntary hospitalizations are far above the national average. Despite that, national research on this field is still scarce. Our study, focusing on factors associated with involuntary hospitalisation in Switzerland, contributes to fill this gap.

The aim of the study was to compare the socio-demographic and clinical profile as well as the referral and hospitalisation process of people voluntarily and involuntarily admitted to four psychiatric hospitals in Switzerland in order to identify which of these factors could predict the use of compulsory admission. A clear identification of the factors associated with coercion could help the local authorities, professionals and researchers to recognise high-risk patients and to promote the development of better-targeted alternative interventions.

## Methods

### Study design and setting

In Switzerland, involuntary admissions are regulated by the Article 426 and 427 of the Swiss Civil Code (CC), which states that a person suffering from mental disorder, mental disability or serious neglect may be committed to an appropriate institution if the required treatment cannot be provided otherwise. A person suffering from a mental disorder who has entered an institution voluntarily and wishes to leave, may be detained by the institution’s medical team for a maximum of 3 days in case of risk to their own life or to the life or the physical integrity of others. At the end of the 3 days period, the patient may leave the institution unless an enforceable hospitalisation is ordered.

At the cantonal level, each canton is responsible for the implementation and specification of the legal framework of involuntary hospitalisation [25].

This retrospective study was based on the analysis of routinely collected data of four psychiatric hospitals in the Canton of Vaud, Switzerland. In this canton, only the doctors designated by the Department of Health and Social Action (DSAS), and the guardianship authority, called Adult Protection Authority (APA), are allowed to order an involuntary hospitalisation. Compulsory admissions ordered by doctors cannot exceed 6 weeks, unless a compulsory order from the Adult Protection Authority is promulgated.

The four hospitals included in the study are the main psychiatric hospitals covering the entire area of the four districts of the Canton of Vaud (3′212.1 Km2), which in 2015 counted a total population of 767′497 people (154′007 in District 1; 190′674 in District 2; 256′789 in District 3; 166′027 in District 4). During the same year, the four psychiatric hospitals provided a total of 0.6 psychiatric beds per 1′000 inhabitants (0.6 in District 1; 0.5 in District 2; 0.7 in District 3; 0.4 in District 4), for an average occupancy rate of 94% (92% in District 1; 90% in District 2; 96% in District 3; 97% in District 4). While District 1 and District 2, respectively covering the east and west regions of the canton, are mixed rural-urban areas, District 3, the central part of the canton, is mainly urban and District 4, in the north, is primarily rural.

All consecutive psychiatric admissions occurred between the 1st January 2015 and the 31st December 2015 were included in the study. Available socio-demographic data included age, gender, marital status, nationality and district of residence. Other socio-demographic variables as living condition and employment status were only partially available and could not be included in the analysis. Clinical characteristics as the main diagnosis, the presence of a secondary diagnosis of addiction and/or personality disorders, and the patients’ mental and social functioning were also available. This latter is regularly assessed at admission by clinicians within the institutions through the 12 observer-rated items of the French validated version of the Health of the Nation Outcome Scale (HoNOS) [26, 27]. An additional item assessing psychotropic medication compliance routinely incorporated to the HoNOS was also included in the study. Item-level scores rather than composite scores were used in the analysis [27].

According to the ICD-10 system, diagnoses were aggregated into broader categories: 1. Organic, including symptomatic, mental disorders (F00-F09); 2. Mental and behavioural disorders due to use of alcohol (F10); 3. Mental and behavioural disorders due to psychoactive substance use (F11-F19); 4. Schizophrenia, schizotypal and delusional disorders (F20-F29); 5. Manic and bipolar affective disorders (F30-F31); 6. Mood [affective] disorders (manic and bipolar affective disorders excluded) (F32-F39); 7. Neurotic, stress-related and somatoform disorders (F40-F48); 8. Behavioural syndromes associated with physiological disturbances and physical factors (F50-F59); 9. Disorders of adult personality and behaviour (F60-F69); 10. Mental retardation (F70-F79); 11. Disorders of psychological development (F80-F89); 12. Behavioural and emotional disorders with onset usually occurring in childhood and adolescence (F90-F98); 13. Other non-mental disorders.

Psychiatric hospitalisations’ patterns during the previous 12 months were also evaluated. Namely, data concerning the presence of at least one psychiatric hospitalisation, the presence of at least one medical involuntary psychiatric hospitalisation (ordered by an authorised doctor), the presence of at least one civil involuntary psychiatric hospitalisation (ordered by the guardianship authority) and the total number of inpatient days during the last 12 months were included in the analysis. Finally, available information about the referral process, the hospital district, the legal status at admission (voluntary or involuntary) and the number of inpatient days were taken into account.

All data were anonymized and the study was approved by the Swiss Ethics Committee on research involving human of Lausanne (05 September 2016; N. 2016–01061).

### Statistical analysis

All statistical tests were two-tailed. To avoid reporting negligible effect sizes because of the large sample size, significance level was set at a conservative *p* < .01. The whole sample was split into two subgroups according to the hospitalisation legal status at admission: namely, voluntary (VH) and involuntary hospitalisation (IH). The associations between legal status at admission and the categorical variables were tested using Pearson’s Chi-square tests. In order to highlight which categories contributed the most to the overall significant result we examined significant standardized residual at the .01 level. Ordinal and continuous variables that did not met assumptions of normality were analysed using Mann-Whitney U test. Independent samples t-tests were otherwise performed.

In order to develop a parsimonious model able to describe the relationship between legal status at admission and the above mentioned variables, a multivariate logistic regression analysis using a stepwise forward likelihood ratio variable selection procedure was performed. The legal status at admission was included as the dependent variable, with voluntary hospitalisation coded as 0 and involuntary hospitalisation coded as 1. Only socio-demographic, clinical and referral variables reaching a *p* < .01 level of significance when comparing the two subgroups were included as independent variables. Psychiatric hospitalisations’ patterns during the previous 12 months were also included into the model, while hospital district and the number of inpatient days were excluded from the analysis as subsequent to the referral process. For categorical variables, the IH group most represented categories were chosen as reference.

All statistical analyses were performed with the IBM SPSS 23.

## Results

Between the 1st January 2015 and the 31st December 2015, 5060 admissions were identified in the four psychiatric hospitals of the Canton of Vaud, Switzerland. Legal status at admission was not available for 33 cases, which were excluded from the analyses. Of the remaining 5027 admissions, 62% were voluntarily hospitalised and 38% were involuntarily hospitalised (Table 1). The whole sample was mainly composed by female, single, with Swiss nationality, an average age of 45 years and a main diagnosis of Schizophrenia, schizotypal and delusional disorders (F20-F29), followed by Mood [affective] disorders (manic and bipolar affective disorders excluded) (F32-F39).

**Table 1:** Socio-demographic characteristics of voluntary and involuntary patients admitted to 4 psychiatric hospitals in Switzerland (*N* = 5027)

Characteristics	VH (*n* = 3109; 61.8%)	IH (*n* = 1918, 38.2%)	*p*-value	Effect size
Demographics				
Age, Mean (SD)	42.5 (16.5)	48.8 (21.5)	<.0001^b^	d = .33^c^
Sex, Male, % (n)	46.5 (1441)	49.9 (957)	.018^a^	V = .03^c^
Marital status, % (n)				
Single	50.7 (1569)	48.3 (918)	<.0001^a^	V = .12^c^
Married/Registered partnership	22.5 (969)	23.3 (443)		
Divorced/Separated	23.5 (728)	19.7 (374)		
Widowed	**3.2 (99)**	**8.8 (167)**		
Nationality				
Swiss, % (n)	65.3 (2018)	65.8 (1254)	.745^a^	V = .01^c^
District of residence, % (n)				
District 1	**19.8 (591)**	**28.1 (517)**	<.0001^a^	V = .10^c^
District 2	22.7 (677)	**17.9 (330)**		
District 3	37.7 (1125)	37.3 (686)		
District 4	19.9 (594)	16.7 (308)		

In bold, cells with significant standardized residual at the .01 level

*VH* Voluntary Hospitalisation, *IH* Involuntary Hospitalisation, *SD* Standard Deviation

^a^ Pearson’s Chi-square. ^b^ Independent samples t-test

^c^Small effect

### Socio-demographic and clinical characteristics

As shown in Table 1, involuntarily admitted patients were significantly older than voluntarily admitted (t(3294.8) = − 11.5; *p* < .0001). Moreover, a significant association was found concerning the marital status (χ^2^(3) = 78.6; *p* < .0001), with more widowed and fewer single and divorced or separated into the IH group compared to the VH group. The analyses showed also a significant association with district of residence (χ^2^(3) = 52.5; *p* < .0001), while no significant results were obtained for gender and nationality.

Clinical characteristics are presented in Table 2. Significant results were found with respect to the main diagnosis. Compared to the VH group, the IH group presented higher percentages of Organic, including symptomatic, mental disorders (F00-F09) (χ^2^(1) = 228.5; *p* < .0001), Schizophrenia, schizotypal and delusional disorders (F20-F29) (χ^2^(1) = 70.8; *p* < .0001), Manic and bipolar affective disorders (F30-F31) (χ^2^(1) = 8.0; *p* = .005), Mental retardation (F70-F79) (χ^2^(1) = 8.2; *p* = .004) and Other non-mental disorders (χ^2^(1) = 10.9; *p* = .001). Conversely, the IH group registered lower percentages of Mood [affective] disorders (manic and bipolar affective disorders excluded) (F32-F39) (χ^2^(1) = 117.4; *p* < .0001), Mental and behavioural disorders due to psychoactive substance use (F11-F19) (χ^2^(1) = 37.1; *p* < .0001) and Neurotic, stress-related and somatoform disorders (F40-F48) (χ^2^(1) = 32.3; *p* < .0001). In addition, the IH group showed a significantly lower percentage of comorbidity for personality disorders (χ^2^(1) = 38.1; *p* < .0001).

**Table 2:** Clinical characteristics of voluntary and involuntary patients admitted to 4 psychiatric hospitals in Switzerland (*N* = 5027)

Characteristics	VH (*n* = 3109)		IH (*n* = 1918)		*p*-value	Effect size
Main diagnosis at discharge (ICD-10), % (n)						
Organic, including symptomatic, mental disorders (F00-F09)	3.0 (89)		14.8 (264)		<.0001^a^	V = .21^d^
Mental and behavioural disorders due to use of alcohol (F10)	10.8 (323)		10.1 (180)		.446^a^	V = .01^c^
Mental and behavioural disorders due to psychoactive substance use (F11-F19)	9.9 (296)		4.9 (88)		<.0001^a^	V = .08^c^
Schizophrenia, schizotypal and delusional disorders (F20-F29)	18.6 (555)		29.1 (518)		<.0001^a^	V = .12^c^
Manic and bipolar affective disorders (F30-F31)	4.8 (142)		6.7 (119)		.005^a^	V = .04^c^
Mood [affective] disorders (manic and bipolar affective disorders excluded) (F32-F39)	23.7 (709)		11.0 (196)		<.0001^a^	V = .15^c^
Neurotic, stress-related and somatoform disorders (F40-F48)	13.7 (408)		8.2 (146)		<.0001^a^	V = .08^c^
Behavioural syndromes associated with physiological disturbances and physical factors (F50-F59)	0.6 (19)		0.3 (6)		.167^a^	V = .02^c^
Disorders of adult personality and behaviour (F60-F69)	11.6 (345)		9.4 (167)		.019^a^	V = .03^c^
Mental retardation (F70-F79)	1.1 (34)		2.2 (39)		.004^a^	V = .04^c^
Disorders of psychological development (F80-F89)	0.8 (24)		0.8 (14)		.949^a^	V = .00^c^
Behavioural and emotional disorders with onset usually occurring in childhood and adolescence (F90-F98)	0.9 (27)		1.0 (17)		.858^a^	V = .00^c^
Other non-mental disorders	0.5 (16)		1.5 (26)		.001^a^	V = .05^c^
Comorbidity at discharge (ICD-10), % (n)						
F10-F19	8.6 (257)		8.9 (159)		.697^a^	V = .01^c^
F60-F69	18.3 (547)		11.6 (206)		<.0001^a^	V = .10^c^
HoNOS scores at admission, Mean (SD) Mdn (IQR)						
Overactive, aggressive, disruptive or agitated behaviour	0.8 (1.2)	0.0 (2.0)	1.7 (1.5)	2.0 (3.0)	<.0001^b^	*r* = −.30^e^
Non-accidental self-injury	0.7 (1.3)	0.0 (1.0)	0.7 (1.2)	0.0 (1.0)	.011^b^	*r* = −.04^c^
Problem drinking or drug-taking	1.2 (1.6)	0.0 (3.0)	1.1 (1.5)	0.0 (2.0)	.006^b^	*r* = −.04^c^
Cognitive problems	0.7 (1.1)	0.0 (1.0)	1.3 (1.4)	1.0 (2.0)	<.0001^b^	*r* = −.18^c^
Physical illness or disability problems	0.7 (1.2)	0.0 (1.0)	1.0 (1.4)	0.0 (2.0)	<.0001^b^	*r* = −.10^c^
Problems associated with hallucinations and delusions	0.7 (1.3)	0.0 (1.0)	1.4 (1.6)	0.0 (3.0)	<.0001^b^	*r* = −.21^d^
Problems with depressed mood	2.3 (1.3)	3.0 (1.0)	1.8 (1.4)	2.0 (3.0)	<.0001^b^	*r* = −.18^c^
Other mental and behavioural problems	2.0 (1.4)	2.0 (2.0)	2.0 (1.4)	2.0 (3.0)	.212^b^	*r* = −.02^c^
Problems with relationships	1.5 (1.2)	2.0 (2.0)	1.8 (1.3)	2.0 (2.0)	<.0001^b^	*r* = −.12^c^
Problems with activities of daily living	1.1 (1.2)	1.0 (2.0)	1.6 (1.4)	2.0 (3.0)	<.0001^b^	*r* = −.16^c^
Problems with living conditions	1.2 (1.4)	1.0 (2.0)	1.3 (1.4)	1.0 (2.0)	.090^b^	*r* = −.03^c^
Problems with occupation and activities	1.6 (1.3)	2.0 (3.0)	1.8 (1.4)	2.0 (3.0)	.0002^b^	*r* = −.06^c^
Problems with psychotropic medication compliance (additional item)	0.7 (1.2)	0.0 (1.0)	1.5 (1.5)	1.0 (3.0)	<.0001^b^	*r* = −.23^d^
At least one psychiatric hospitalisation during the last 12 months, % (n)	45.0 (1405)		34.7 (666)		<.0001^a^	V = .10^c^
At least one medical involuntary psychiatric hospitalisation during the last 12 months, % (n)	13.9 (431)		22.1 (424)		<.0001^a^	V = .11^c^
At least one civil involuntary psychiatric hospitalisation during the last 12 months, % (n)	1.1 (34)		4.1 (79)		<.0001^a^	V = .10^c^
Number of inpatient days during the last 12 months, Mean (SD) Mdn (IQR)	20.0 (36.2)	0.0 (26.8)	16.4 (37.6)	0.0 (14.9)	<.0001^b^	*r* = −.10^c^

*VH* Voluntary Hospitalisation, *IH* Involuntary Hospitalisation, *SD* Standard Deviation, *Mdn* median, *IQR* interquartile range

^a^ Pearson’s Chi-square. ^b^ Mann-Whitney U Test

^c^Small effect ^d^Medium effect ^e^Large effect

The comparison of the two subgroups on the HoNOS item-level scores showed a significant difference on 9 items out of 12. Namely, the IH group scored higher on Overactive, aggressive, disruptive or agitated behaviour (U = 1,630,784.5; z = − 20.3; *p* < .0001), Cognitive problems (U = 1,807,241.5; z = − 11.9; *p* < .0001), Physical illness or disability problems (U = 2,186,339.0; z = − 5.4; *p* < .0001), Problems associated with hallucinations and delusions (U = 1,814,924.5; z = − 14.0; *p* < .0001), Problems with relationships (U = 1,851,882.5; z = − 8.0; *p* < .0001), Problems with activities of daily living (U = 1,755,666.0; z = − 10.6; p < .0001) and Problems with occupation and activities (U = 1,921,846.0; z = − 3.6; *p* = .0002). A significantly higher score was also reached by the involuntarily hospitalised patients on the HoNOS additional item Problems with psychotropic medication compliance (U = 1,478,617.5; z = − 15.0; *p* < .0001). Conversely, the IH group scored significantly lower on Problem drinking or drug-taking (U = 2,109,601.0; z = − 2.8; *p* = .006) and Problems with depressed mood (U = 1,854,626.5; z = − 11.8; *p* < .0001).

Finally, only 34.7% of involuntarily hospitalised patients had at least one psychiatric hospitalisation during the previous year, compared to 45.0% of the voluntarily hospitalised (χ^2^(1) = 53.6; *p* < .0001). Furthermore, the average number of inpatient days during the last 12 months was significantly smaller within the IH group compared to the VH group (U = 2,667,779.5; z = − 7.0; *p* < .0001). Conversely, within the IH group 22.1% of patients had at least one medical involuntary hospitalisation and 4.1% had at least one civil involuntary hospitalisation, compared respectively to only 13.9% and 1.1% within the VH group (χ^2^(1) = 57.1, *p* < .0001; χ^2^(1) = 49.4, *p* < .0001).

### Referral and hospitalisation process characteristics

Referral and hospitalisation process characteristics are presented in Table 3. With respect to the referral process, significant results emerged comparing the two subgroups. Committed patients were more likely referred by a general practitioner (χ^2^(1) = 125.6; *p* < .0001), a general hospital (χ^2^(1) = 43.4; *p* < .0001), a psychiatric hospital (χ^2^(1) = 56.4; *p* < .0001), the civil justice or other justice authority (χ^2^(1) = 74.1; *p* < .0001) and the police (χ^2^(1) = 15.9; *p* < .0001). Only 2.1% of involuntary patients were auto referred (χ^2^(1) = 253.9; *p* < .0001) and only 33.8% were referred by an ambulatory psychiatrist (χ^2^(1) = 74.5; *p* < .0001).

**Table 3:** Referral and hospitalisation process of voluntary and involuntary patients admitted to 4 psychiatric hospitals in Switzerland (*N* = 5027)

Characteristics	VH (*n* = 3109)		IH (*n* = 1918)		*p*-value	Effect size
Referral from, % (n)						
Patient	17.3 (512)		2.1 (38)		<.0001^a^	V = .23^d^
Family/relatives	2.5 (73)		1.5 (27)		.023^a^	V = .03^c^
General practitioner	11.3 (335)		23.6 (426)		<.0001^a^	V = .16^c^
General hospital	15.3 (452)		22.9 (413)		<.0001^a^	V = .10^c^
Ambulatory psychiatrist	46.5 (1375)		33.8 (610)		<.0001^a^	V = .12^c^
Psychiatric hospital	4.1 (121)		9.5 (171)		<.0001^a^	V = .11^c^
Other institution	1.8 (54)		1.8 (33)		.997 ^a^	V = .00^c^
Civil justice/ other justice authority	0.1 (2)		2.7 (49)		<.0001^a^	V = .13^c^
Police	0.5 (15)		1.7 (30)		<.0001^a^	V = .06^c^
Other	0.6 (19)		0.5 (9)		.528 ^a^	V = .01^c^
Hospital District, % (n)						
District 1	**18.5 (576)**		**24.6 (472)**		<.0001^a^	V = .09^c^
District 2	25.5 (794)		**20.4 (391)**			
District 3	37.7 (1172)		39.1 (750)			
District 4	18.2 (567)		15.9 (305)			
Number of inpatient days, Mean (SD) Mdn (IQR)	21.2 (25.3)	14.3 (19.0)	33.1 (40.9)	21.0 (32.5)	<.0001^b^	*r* = −.16^c^

In bold, cells with significant standardized residual at the .01 level

*VH* Voluntary Hospitalisation, *IH* Involuntary Hospitalisation, *SD* Standard Deviation, *Mdn* median, *IQR* interquartile range

^a^ Pearson’s Chi-square. ^b^ Mann-Whitney U Test

^c^Small effect ^d^Medium effect

Concerning the hospital district, significant variations were found on involuntary hospitalisation rates, ranging from 45% of total admissions in District 1 to 33% in District 2 (χ^2^(3) = 38.7; *p* < .0001).

Finally, compulsory hospitalisations were significantly longer than voluntary hospitalisations, with on average 33.1 ± 40.9 inpatient days for the IH group and 21.2 ± 25.3 for the VH group (U = 2,407,984.5; z = − 11.3; *p* < .0001).

### Factors associated with involuntary hospitalisations

The multivariate logistic regression model to test factors associated with involuntary hospitalisation is presented in Table 4 (see Additional file 1 for further details about the stepwise selection procedure).

**Table 4:** Factors associated with involuntary hospitalisations to 4 psychiatric hospitals in Switzerland: final model (*N* = 2965)

Predicting factors	B (S.E.)	OR	95% C.I.		*p*-value
Main diagnosis (ref. F20-F29):					
Organic, including symptomatic, mental disorders (F00-F09)	0.731 (0.210)	2.077	1.376	3.137	.001
Mental and behavioural disorders due to psychoactive substance use (F11-F19)	−0.583 (0.206)	0.558	0.372	0.836	.005
HONOS items:					
Overactive, aggressive, disruptive or agitated behaviour	0.315 (0.037)	1.370	1.275	1.473	<.0001
Problems associated with hallucinations and delusions	0.115 (0.040)	1.122	1.037	1.214	.004
Problems with depressed mood	−0.165 (0.039)	0.848	0.786	0.915	<.0001
Problems with psychotropic medications compliance (additional item)	0.179 (0.036)	1.196	1.114	1.283	<.0001
Referred from (ref. Ambulatory psychiatrist):					
General practitioner	0.877 (0.130)	2.405	1.864	3.102	<.0001
General hospital	0.731 (0.126)	2.078	1.623	2.660	<.0001
Psychiatric hospital	0.912 (0.200)	2.490	1.683	3.684	<.0001
Patient	−1.438 (0.225)	0.238	0.153	0.369	<.0001
District of residence (ref. District 1):					
District 2	−0.618 (0.139)	0.539	0.411	0.707	<.0001
District 3	−0.352 (0.114)	0.704	0.563	0.879	.002
District 4	−0.636 (0.150)	0.529	0.394	0.711	<.0001
Psychiatric hospitalisation during the last 12 months	−0.738 (0.135)	0.478	0.367	0.623	<.0001
Involuntary medical hospitalisation during the last 12 months	1.190 (0.146)	3.287	2.468	4.377	<.0001
Involuntary civil hospitalisation during the last 12 months	1.710 (0.328)	5.531	2.909	10.517	<.0001
Number of inpatient days during the last 12 month	−0.005 (0.002)	0.995	0.992	0.999	.002
Intercept	−0.690 (0.182)	0.502			<.0001

*C.I* Confidence Interval, *OR* Odds Ratio

χ^2^(32) = 885.5; *p* < .0001; Nagelkerke R^2^ = .36

People affected by Organic mental disorders (F00-F09) were more likely to be coerced compared to people affected by Schizophrenia, schizotypal and delusional disorders (F20-F29). Conversely, patients affected by Mental and behavioural disorders due to psychoactive substance use (F11-F19) were less likely to be involuntary hospitalised compared to patients with a main diagnosis of Schizophrenia, schizotypal and delusional disorders (F20-F29).

Four HoNOS items were associated with involuntary hospitalisation. Higher scores on Overactive, aggressive, disruptive or agitated behaviour, Problems associated with hallucinations and delusions, and on the HoNOS additional item Problems with psychotropic medication compliance were associated with a higher risk of compulsion. Vice versa, the probability of being involuntarily hospitalised decreased significantly with increased depressed mood.

In addition, the model revealed that patients referred by a general practitioner, a general hospital and a psychiatric hospital presented a higher probability of compulsory admission compared to patients referred by an ambulatory psychiatrist. On the contrary, auto-referred patients were less likely involuntarily hospitalised.

With respect to patients’ socio-demographic characteristics, the analysis showed that living in any district but District 1 was associated with a significantly reduced risk of involuntary hospitalisation, especially for District 2 and District 4.

Finally, being hospitalised at least once during the last 12 months was associated with a reduced risk of compulsory admission, as well as having a higher number of inpatient days during the previous year. On the contrary, being involuntarily hospitalised at least once during the last year was the variable most strongly associated with a further use of coercion. Precisely, the probability of being involuntarily admitted increased by a factor of 3.2 if the patient had at least one previous involuntary medical hospitalisation and by a factor of 5.5 if they had at least one previous involuntary civil hospitalisation.

Globally, the model explained 36% of the variance.

## Discussion

The main aim of this paper was to compare the socio-demographic and clinical profile as well as the referral and hospitalisation process of people voluntarily and involuntarily admitted to four psychiatric hospitals in Switzerland, in order to identify which of these factors could be associated with the use of compulsory admission.

Results showed that the voluntary and involuntary subgroups differed significantly on several variables.

Both, individual and system-related factors were associated with compulsory admission. People affected by organic mental disorders (F00-F09), with higher levels of psychotic symptoms, aggression, and problems with medication adherence, were more likely to be involuntarily admitted. Moreover, living in District 1, being referred by a general practitioner, a general hospital or a psychiatric hospital instead of an ambulatory psychiatrist and being involuntarily hospitalised at least once during the previous 12 months, were associated with an increased probability of coercion. Conversely, auto-referred patients affected by Mental and behavioural disorders due to psychoactive substance use (F11-F19), with higher levels of depression were less likely to be committed.

These results confirmed partially what previously suggested by the international literature about the factors associated with coercion.

In several previous studies, schizophrenia was identified as the main diagnosis predicting compulsion [1, 10, 11, 13, 15, 16]. In our model, the diagnosis of psychotic disorder was confirmed as more strongly associated with coercion only by comparison to the diagnosis of Mental and behavioural disorders due to psychoactive substance use (F11-F19). People affected by organic mental disorders (F00-F09) showed the highest odds of involuntary admission, confirming what already emerged in a Danish study on risk factors of coercive measures [12]. Further studies should focus on the complex needs of this specific population and the development of new alternative treatments. Between 2013 and 2017, in District 3 the number of beds for people aged above 65 years decreased from 80 to 50. This closure of beds was followed by the development of psychogeriatric mobile teams, which aim to provide home treatment as alternative to hospitalisation. The effectiveness of this pilot project is still under assessment, but results seem promising.

Furthermore, higher levels of aggression and psychotic symptoms increased the odds of coercion. Aggressive behaviours can often require the intervention of the police during the admission process, which in a previous study was found to be associated with an increased risk of compulsion [18]. However, these results could also be partially explained by the growing social intolerance to certain deviant or inappropriate behaviours, which strengthen the need of control and containment [28]. Previous studies have found that the perceived risk of danger to self or others is determinant in professionals’ decisions about involuntary admissions [29, 30]. Besides the application of a danger-criterion, the use of coercion seems to be also justified by the will to preserve the patients’ health and safety. People with lower medication compliance were more likely to be coerced.

At the individual level, the only socio-demographic variable associated with coercion was the district of residence. Living in any district but District 1 decreased the odds of being involuntarily hospitalised. This result confirmed the existence of important variations not only at the international [8] or intercantonal level [11, 22, 24], but also at the district level. In 2015, 38.2% of the total admissions to the four psychiatric hospitals of the Canton of Vaud were involuntary, ranging from 33% of District 2 to 45% of District 1. Many assumptions could be made to explain these variations, such as the existence of significant differences in the population profiles and state of mental health of each district. Epidemiological comparative studies should be promoted in order to verify this hypothesis.

Moreover, previous studies proved the association between several area-related characteristics, such as socio-economic deprivation, ethnic density and urbanicity level, and the use of coercion [14, 21, 23]. Out of the scope of the present study, this hypothesis should be further investigated in future researches.

Even though all districts were subjected to the same regulation, important differences existed in terms of mental health services’ provision. Several studies have tried to explain the association between the offer of psychiatric beds and the use of compulsory admission, but this link remains unclear [20–22]. In our study, the district with the highest rate of involuntary hospitalisation (District 1) had also the second highest number of psychiatric beds per 1′000 inhabitants (0.6 in District 1; 0.5 in District 2; 0.7 in District 3; 0.4 in District 4) and the second lowest average occupancy rate (92% in District 1; 90% in District 2; 96% in District 3; 97% in District 4).

Concerning the outpatient services, since 2001 Assertive Community Treatment teams have been implemented in the Canton of Vaud [31]. When sufficiently provided, these teams may represent valid alternatives to hospitalisation, even under compulsion. However, in the Canton of Vaud mobile psychiatry teams are little developed and large disparities exist between the different districts of the canton for this offer of care, which could partially explain the different involuntary hospitalisation rates.

Concerning the association between referral process and compulsory admission, our results confirmed what already suggested in previous studies [11]. People referred by a general practitioner, a general hospital and a psychiatric hospital had more than two-fold elevated odds of being committed compared to someone referred by an ambulatory psychiatrist. To better understand this result, two important considerations should be taken into account. Firstly, until 2016 only general practitioners provided out-of-hours psychiatric home care in the Canton of Vaud. Secondly, since in some districts psychiatric emergency services are not developed, patients with mental health crises are often evaluated in general medical emergency services. These evaluations are mainly provided in difficult conditions by professionals who do not know the patient, which make finding an alternative to an involuntary admission more complex [18, 32]. The fact that, in an area where the rate of psychiatrists per inhabitants is one of the highest in the world (0.73 psychiatrists per 1′000 inhabitants in 2015) and psychiatric services are highly developed, most of the involuntarily admitted patients are referred by doctors who do not know them, questions the follow up of people with severe mental disorders and the ability to prevent hospitalisations.

Finally, according to our results the variable most strongly associated with commitment was coercion itself. People with at least one involuntary medical or civil admission during the previous year seemed to have respectively more than three-fold and five-fold elevated probability of being coerced one more time. On the contrary, being hospitalised at least once during the last 12 months was associated with a halved likelihood of a new involuntary hospitalisation. This observation can be seen as tautological, as some of the characteristics of the patients placed under compulsion are stable. Moreover, even though this result confirmed what already emerged in previous studies [10, 33–35], the underlying process remains unclear. However, it seems possible to reduce the iterative use of coercion by adapting models of care. For example, Van der post et al. [36] found that increasing patients’ satisfaction with care, especially for patients involuntarily admitted, could reduce the risk of new involuntary hospitalisation.

During the last decades, great efforts have been made worldwide to promote the development of alternative forms of intervention able to reduce the use of coercion in psychiatry. In 2016, a meta-analysis aiming to establish which interventions could effectively reduce compulsory admissions in adult psychiatric patients showed that advanced statements decreased significantly the risk of coercion [37]. This result emphasises the need of tools that reinforce shared decision-making, such as Joint Crisis Plan [38], to deal with crisis situations. Other models of intervention, like Crisis Resolution Teams (CRTs) [39, 40], aimed to reduce the admission rates of people suffering from acute mental crisis, but evidence on their effectiveness is still limited. Moreover, the CTRs main effect concerned voluntary hospitalisations, while the risk of readmission for people previously involuntarily hospitalised seemed to increase despite this intervention [41]. More recently, an innovative programme, addressing patients’ self-management skills and combining individualised psychoeducation, crisis cards and long-term (24 months) preventive monitoring, was implemented and tested in Zurich, Switzerland [42]. First results seemed promising, reducing compulsory readmission and the length of involuntary episodes [35], but replication studies are mandatory to confirm these findings.

This study has some limitations. Since the study was based on the analysis of routinely collected data, only few variables were available on the referral and hospitalisation process. Another limitation is that in some instance stepwise regression may not be adequate and capitalize on the chance characteristics of the data (e.g. increasing type 1 error). Given our large sample size, we wanted to build a parsimonious model and therefore we adopted a more stringent entry criteria (*p* < .01). We consider that this restrictive approach overcome potential problems with variable selection procedures. Furthermore, sensitivity analysis with another variable selection procedure (backward likelihood ratio) showed identical results.

## Conclusion

Involuntary hospitalisation rates in Switzerland, and even more in the Canton of Vaud, are among the highest in Europe. Because of its proved strong negative effects on patients, it is a paramount goal of the local authorities to limit as much as possible the recourse to this practice. The present study identified several individual-related, as well as system-related factors associated with the use of coercion. These results allowed us to trace a clear profile of high-risk patients and to provide several inputs that could help local authorities, professionals and researchers to develop better-targeted alternative interventions reducing the use of coercion.

## Additional files

Additional file 1: Logistic regression: stepwise selection procedure and changes. (DOCX 23 kb)

## Abbreviations

HoNOS: Health of the Nation Outcome Scale
IH: Involuntary hospitalisation
VH: Voluntary hospitalisation
