# Supplementary material for: Factors associated with involuntary hospitalisation for psychiatric patients in Switzerland: a retrospective study
Source: BMC Psychiatry. 2018 Dec 29;18:401. doi: 10.1186/s12888-018-1966-6 (PMC6311042; doi:10.1186/s12888-018-1966-6)
Supplement: Supplementary file 1 — Logistic regression: stepwise selection procedure and changes. (DOCX 23 kb) [file 12888_2018_1966_MOESM1_ESM.docx]

| **Additional file 1**  **Logistic regression: stepwise selection procedure and changes** | | | | | | | | | | | | |
| --- | --- | --- | --- | --- | --- | --- | --- | --- | --- | --- | --- | --- |
|  |  |  | **Chi-Square** | | |  | **-2 Log Likelihood** | | |  | **R squared equivalent** | |
| **Step** | **Variables added** |  | **Value** | **df** | **p-value** |  | **Change** | **df** | **p-value** |  | **Cox & Snell R^2^** | **Nagelkerke R^2^** |
| 1 | Overactive, aggressive, disruptive or agitated behaviour |  | 289.5 | 1 | <.0001 |  | 289.5 | 1 | <.0001 |  | .09 | .13 |
| 2 | Referred from |  | 594.3 | 10 | <.0001 |  | 304.7 | 9 | <.0001 |  | .18 | .25 |
| 3 | Main diagnosis |  | 683.6 | 22 | <.0001 |  | 89.3 | 12 | <.0001 |  | .21 | .28 |
| 4 | Problems with psychotropic medications compliance (additional item) |  | 717.6 | 23 | <.0001 |  | 33.9 | 1 | <.0001 |  | .22 | .29 |
| 5 | District of residence |  | 754.3 | 26 | <.0001 |  | 36.7 | 3 | <.0001 |  | .23 | .31 |
| 6 | Involuntary medical hospitalisation during the last 12 months |  | 779.3 | 27 | <.0001 |  | 25.1 | 1 | <.0001 |  | .23 | .32 |
| 7 | Psychiatric hospitalisation during the last 12 months |  | 828.3 | 28 | <.0001 |  | 48.9 | 1 | <.0001 |  | .24 | .34 |
| 8 | Involuntary civil hospitalisation during the last 12 months |  | 851.5 | 29 | <.0001 |  | 23.2 | 1 | <.0001 |  | .25 | .34 |
| 9 | Problems with depressed mood |  | 869.8 | 30 | <.0001 |  | 18.3 | 1 | <.0001 |  | .25 | .35 |
| 10 | Problems associated with hallucinations and delusions |  | 877.6 | 31 | <.0001 |  | 7.8 | 1 | .005 |  | .26 | .35 |
| 11 | Number of inpatient days during the last 12 month |  | 885.5 | 32 | <.0001 |  | 7.8 | 1 | .005 |  | .26 | .35 |
